# Multidisciplinary Team Perceptions on Preparing Newborns for Discharge From a Rooming‐in Unit: A Qualitative Study

**DOI:** 10.1002/hsr2.72422

**Published:** 2026-04-27

**Authors:** Thaís Emanuele da Conceição, Adriana Duarte Rocha, Cristina Lumia, Simona Fumagalli, Antonella Nespoli, Maria Helena do Nascimento Souza

**Affiliations:** ^1^ Department of Public Health, Anna Nery School of Nursing Universidade Federal do Rio de Janeiro Rio de Janeiro Brasil; ^2^ Instituto Nacional da Saúde da Mulher, da Criança e do Adolescente Fernandes Figueira–Fundação Oswaldo Cruz Unidade de Pesquisa Clínica Rio de Janeiro Brazil; ^3^ School of Medicine and Surgery University of Milano‐Bicocca Monza Italy

## Abstract

**Background:**

Rooming‐in is recommended by the World Health Organization as part of essential newborn care and plays a key role in promoting maternal‐infant bonding, breastfeeding, and continuity of care from hospital to home. Despite evidence supporting multidisciplinary discharge planning and parental preparation, the discharge process of newborns remains complex and may be influenced by organizational structures, professional roles, and communication practices within healthcare teams. Limited evidence is available on how multidisciplinary teams perceive and manage newborn discharge preparation in rooming‐in settings.

**Aim:**

To explore the perspectives of a multidisciplinary team engaged in the preparation of newborns for discharge from a rooming‐in facility. Specifically, this study aims to identify the experiences of Brazilian healthcare professionals to improve the entire transition of care from hospital to home.

**Methods:**

A qualitative study took place in Brazil from January to October 2020. Nineteen healthcare professionals participated in this research. Semi‐structured interviews were conducted following the framework methodology. Data were analyzed using inductive thematic analysis.

**Findings:**

Three themes were identified from the data extracted and conceptualized for showing the critical moment of discharging a newborn: (1) Responsibility on the discharge of women and newborns from the hospital; (2) Leadership and shared information about healthcare practices; (3) Transition from hospital care to care at home. One sub‐theme emerged concerning breastfeeding.

**Conclusion:**

Findings highlight that the discharge process of the mother‐infant dyad is largely perceived as physician‐led, with limited interdisciplinary involvement in decision‐making and parental education. This gap underscores the need to strengthen multidisciplinary collaboration and to establish standardized discharge procedures to ensure comprehensive and consistent parental counseling.

## 1 Introduction

Rooming‐in is a practice whereby newborns are allowed to stay in the same room as mothers for the duration of their hospital stay, beginning immediately after birth [1]. The World Health Organization (WHO) recommends rooming‐in within its essential newborn care guidelines, recognizing its significance in fostering maternal‐infant bonding and enhancing health outcomes for both individuals [2]. Rooming‐in facilitates caring by allowing women to more promptly respond to their infants' feeding cues, therefore promoting effective breastfeeding habits [3]. Implementing extensive training and complying with WHO guidelines, particularly in limited‐resource environments, can markedly enhance infant care and diminish mortality rates [4]. The rooming‐in of the parent‐infant dyad is a nonpharmacological intervention linked to improved hospital outcomes for newborns, highlighting the necessity for sustained parental participation and beneficial effects on postdischarge transitions [5]. Within this model, discharge preparation is not limited to a single event but is progressively constructed through continuous interaction between healthcare professionals and families during hospitalization.

Evidence supporting rooming‐in emphasizes its importance not only during hospitalization but also as a component of efficient discharge preparation [6]. For instance, studies highlight the significance of personalized transitional care plans and evaluating discharge preparation in newborns and their parents to prevent unplanned readmissions [7]. Newborns are often readmitted within 30 days due to problems such as sickness and nutrition difficulties associated with breastfeeding. The imperative for healthcare professionals is to enhance that parents obtain comprehensive knowledge concerning post‐discharge care [8]. The WHO has created a Predischarge Checklist for Healthy Term Newborns to assist professionals in facilitating proper discharge procedures. However, the discharge of newborns is a multidisciplinary procedure [9].

A collaborative approach involving multiple healthcare professionals is essential in the discharge planning process, as it mitigates potential risks and empowers families for care at home [10]. The collaboration of different professionals—such as doctors, nurses, midwives, and social workers—promotes that all facets of newborn care are thoroughly handled [11]. Nursing educational interventions, such as creating instructional videos about newborn bathing, empower parents with essential skills for caring for their infants [12]. Otherwise, midwives are also essential to ensuring continuity of care posthospital discharge in home visiting and community settings [13].

## 2 Background

The Brazilian Ministry of Health (Ministério da Saúde—MS) defines a newborn as an infant aged 0–28 days [14], characterized by specific health‐related needs due to vulnerability and dependence on care from birth through the transition to home and the post‐discharge period [15]. In Brazil, post‐neonatal mortality rates have seen substantial declines in recent years, attributed to improvements in socio‐environmental and economical situations [16], along with innovations in healthcare techniques. Nonetheless, evidence pertaining to infant mortality necessitates additional investigation and intervention, emphasizing the importance of healthcare service [16, 17].

Following the birth of the newborn, it is crucial for the local healthcare team in the inpatient unit—be it the Neonatal Intensive Care Unit (NICU), Conventional Intermediate Neonatal Care Unit (UCINCo), Kangaroo Intermediate Neonatal Care Unit (UCINCa), or Rooming‐In Unit (RIU)—to deliver care tailored to the requirements of the mother‐infant dyad and the family before hospital discharge, thereby facilitating safe and effective preparation for the home environment [18].

Several studies demonstrate that the organization of follow‐up care should be centered on a strong connection between the professionals and families, marked by effective communication within the multidisciplinary team and clear, direct guidance that encourages safety and trust [19]. However, hospital discharge is a complex and dynamic procedure that necessitates a localized investigation. Despite increasing attention to discharge planning and continuity of care in neonatal settings, there is a lack of evidence exploring how discharge preparation is concretely experienced and enacted within RIUs. In particular, the perspectives of multidisciplinary healthcare teams and the ways in which professional roles, responsibilities, and communication practices shape discharge processes in this context remain underexplored.

### 2.1 Aim

The aim of the study is to explore healthcare professionals' perceptions of the discharge process in the RIU according to a Brazilian setting. Specifically, this study sought to investigate strategies that could improve the transition from hospital to home.

## 3 Methods

### 3.1 Study Design

A qualitative descriptive approach was used for this study. Qualitative description refers to a frequently used expression for qualitative research related to health care and nursing phenomena [20]. The study was conducted through semi‐structured [21], recorded, and face‐to‐face interviews with study participants who agreed to take part in the research.

The Consolidated Criteria for Reporting Qualitative Research checklist was used as a guide for reporting a qualitative study.

### 3.2 Conceptual Framework

This study was informed by established conceptual perspectives on care transitions, interprofessional collaboration, and responsibility in discharge planning, which guided the interpretation of the data.

The concept of transitions of care frames discharge as a complex and critical phase requiring coordination, continuity, and effective communication across healthcare settings, particularly from hospital to home [22]. This perspective emphasizes the need for structured processes and shared responsibility among healthcare professionals to ensure safe transitions. In addition, interprofessional collaboration frameworks were considered to explore how roles, communication, and decision‐making are distributed within multidisciplinary teams. These frameworks highlight the importance of shared goals, role clarity, and coordinated practices in delivering effective care.

Furthermore, the concept of discharge readiness was used as a complementary lens to understand how preparedness for discharge is shaped by multiple dimensions, including physical, psychological, educational, and organizational factors [23].

These conceptual perspectives were not used deductively to generate codes but rather to support the interpretation and contextualization of the findings within the broader literature.

The Framework Method was applied as a systematic analytic approach for managing and analyzing qualitative data.

### 3.3 Study Setting and Recruitment

The study was carried out at a RIU of a Brazilian federal facility associated with the Unified Health System, situated in Rio de Janeiro. Although the hospital specializes in high‐complexity neonatal care, the RIU primarily accommodates clinically stable term newborns and selected late preterm infants who no longer require intensive or intermediate care. Newborns admitted to the RIU may include infants with chronic or uncommon conditions who are clinically stable and whose care can be safely managed alongside their mothers. Examples include newborns with controlled metabolic conditions, mild congenital anomalies not requiring immediate intervention, feeding difficulties under clinical monitoring, or infants recovering from medical or surgical conditions after completion of the acute phase of care. Newborns requiring ongoing intensive monitoring, invasive ventilation, or immediate postoperative care are managed in the NICU or intermediate care units and are transferred to the RIU only once stabilized.

The study period overlapped with the COVID‐19 pandemic, which significantly impacted healthcare systems in Brazil. This context may have influenced care organization, staffing, and discharge practices within the RIU.

The unit infrastructure comprised 14 inpatient beds, including four allocated for urgent postpartum care and ten for late postpartum care. All beds were separated by polyvinyl chloride (PVC) barriers and featured plastic curtains at the front. Two medical offices were available: one for obstetricians and one for neonatologists. The nursing team's station was located at the center of the unit, allowing continuous visual oversight of the beds.

Study participants were a cohort of neonatologists, nurse‐midwives, and nursing technicians. Participants were directly invited through written invitations. Those who expressed willingness to participate were subsequently enrolled in the study. Professionals were selected using a purposive sampling strategy [24] aimed at capturing in‐depth perspectives from professionals routinely involved in discharge preparation. Professionals whose involvement was intermittent or referral‐based were not included, as their perspectives would not reflect the everyday discharge practices explored in this study.

The principal investigator (T.E.) explained the study aims and procedures during the recruitment phase. The researcher introduced herself and the study both during recruitment and again at the beginning of each interview. Following written informed consent, interviews were scheduled at a later time based on participants' availability. All invited participants who agreed to participate completed the study, and no withdrawals occurred.

### 3.4 Inclusion and Exclusion Criteria

Neonatologist, nurse‐midwives and nursing technicians participating in the discharge process were included in this study. No limitations were placed on the demographic variables or professional experiences of the participants. Principal investigator (T.E.) had no past relationship with the Department of professionals interviewed.

### 3.5 Data Collection

All interviews were conducted in Portuguese, the native language of participants, and were transcribed verbatim in Portuguese. Selected excerpts included in the manuscript were translated into English during the analysis and manuscript preparation phase. To ensure translation accuracy, translations were reviewed by bilingual members of the research team and discussed among researchers to preserve the original meaning of participants' accounts.

Interviews were audio recorded and transcribed for each participant by one researcher (T.E). An interview guide was developed based on a review of the existing literature [18, 19, 21, 25] on newborn discharge preparation, continuity of care, and parental counseling, as well as established methodological guidance for qualitative semi‐structured interviews. Conceptual frameworks were used to ensure content relevance and consistency across interviews, while allowing flexibility to explore participants' experiences in depth [25]. The interview guide explored key domains, including discharge preparation practices, professional roles and responsibilities, communication within the multidisciplinary team, parental education, and the transition from hospital to home.

One author (T.E.) conducted two pilot interviews to ensure the clarity and applicability of the questions; the participants were informed of the purpose of their contribution and given the option to suggest any improvements. The interview guide remained unchanged. The final version of the interview guide is provided as supporting Material.

The data from pilot interviews were excluded from the final data analysis. Senior researchers (A.R., M.H.) from Brazil reviewed the interview guide to ensure that it was culturally appropriate and capable of sufficiently exploring the research topic.

Subsequently, individual semi‐structured interviews were conducted from January 2020 to October 2020. All interviews were conducted in person and digitally recorded. No one else was present during the interviews. No interviews were repeated; however, all participants received a transcript of their interview for comment or correction. Field notes were taken during the interviews and used to support data interpretation. Data saturation was determined through ongoing team discussions during data collection and analysis [26]. After 19 interviews, no new codes or themes emerged, and additional data were considered unlikely to provide further meaningful insights.

### 3.6 Data Analysis

An inductive thematic analysis approach was applied [27]. Interview recordings were transcribed utilizing Microsoft Word. To ensure security and privacy of personal data, anonymization was set up for participants by assigning a code (D for doctors, MID for nurse‐midwives, and NUR for nursing technicians).

**Figure 1:**
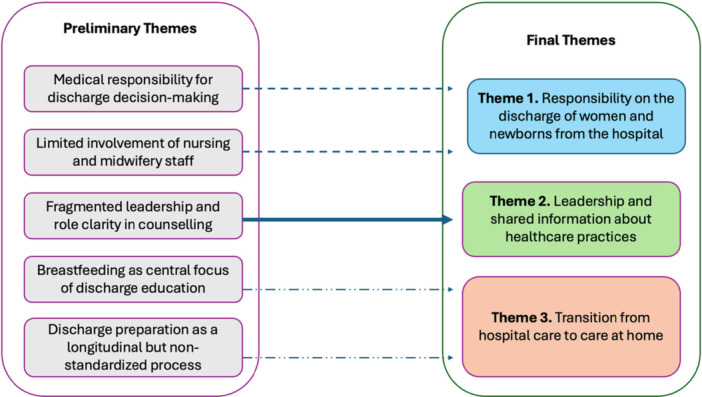
Thematic analysis process and consolidation of themes. This figure illustrates the analytic process used in this qualitative study, showing how five preliminary themes identified during the initial coding phase were iteratively reviewed, compared, and consolidated into three final themes. Preliminary themes emerged from inductive coding of semi‐structured interviews conducted with healthcare professionals working in a rooming‐in unit. Through team discussion and consensus using the Framework Method, conceptually overlapping themes were merged into three final themes: (1) Responsibility on the discharge of women and newborns from the hospital, (2) Leadership and shared information about healthcare practices (with breastfeeding retained as a sub‐theme), and (3) Transition from hospital care to care at home.

Data analysis was performed using Microsoft Excel, involving three researchers (T.E., A.R., M.H.) independently. Relevant terms and quotes were highlighted as code. All codes were aggregated within the same cells to identify five preliminary themes. Patterns in the dataset were analyzed during a group briefing. In certain essential instances, analyses were undertaken many times or through repeated readings. After additional briefing, the five themes were consolidated into three in a process of findings interpretation (Figure 1). Participants provided feedback on the final findings.

### 3.7 Reflexivity

Researcher reflexivity was considered throughout the study. The multidisciplinary background of the research team (midwifery, nursing, and public health) may have influenced data collection and interpretation. To address this, regular team discussions were held to reflect on potential assumptions and to support a balanced and critical interpretation of the data.

### 3.8 Ethical Consideration

This study complied with Resolution No. 466/12 [28] and was approved by the Research Ethics Committee (CEP) of the National Institute for Women's, Children's and Adolescents' Health Fernandes Figueira of the Oswaldo Cruz Foundation (IFF/FIOCRUZ), under opinion number 3.098.916, CAAE: 04636818.9.0000.5269.

To protect participant confidentiality, professional roles were reported in an aggregated manner, and potentially identifiable details were minimized in both the presentation of results and the use of quotations.

## 4 Findings

### 4.1 Characteristics of Participants

Nineteen healthcare professionals were sampled in the Rooming‐in Department. Five doctors: one senior neonatologist, four neonatologists; Five nurse‐midwives and nine professionals from the nursing staff. Table 1 defines the personal and professional details of each participant. No one refused to participate or dropped out.

**Table 1:** Characteristics of study participants.

Code	Role	Age range	Education	Duration of interviews (min)
D01	Senior Neonatologist	55–65	Post‐graduate	16
D02	Neonatologist	30–35	Post‐graduate	22
D03	Neonatologist	30–35	Post‐graduate	19
D04	Neonatologist	35–45	Post‐graduate	23
D05	Neonatologist	35–45	Post‐graduate	29
MID01	Nurse‐Midwife	35–45	Bachelor's degree	36
MID02	Nurse‐Midwife	35–45	Bachelor's degree	38
MID03	Nurse‐Midwife	35–45	Bachelor's degree	40
MID04	Nurse‐Midwife	35–45	Bachelor's degree	33
MID05	Nurse‐Midwife	45–50	Bachelor's degree	31
NUR01	Nursing staff	45–50	Diploma	19
NUR02	Nursing staff	45–50	Diploma	17
NUR03	Nursing staff	45–50	Diploma	15
NUR04	Nursing staff	55–60	Diploma	17
NUR05	Nursing staff	55–60	Diploma	15
NUR06	Nursing staff	55–60	Diploma	21
NUR07	Nursing staff	45–50	Diploma	27
NUR08	Nursing staff	45–50	Diploma	21
NUR09	Nursing staff	45–50	Diploma	23

The identified main themes were: (1) Responsibility on the discharge of women and newborns from the hospital; (2) Leadership and shared information about healthcare practices; (3) Transition from hospital care to care at home. One sub‐theme emerged concerning breastfeeding.

### 4.2 Responsibility on the Discharge of Women and Newborns From the Hospital

This theme reflects participants' perceptions of a predominantly physician‐led discharge process, highlighting limited involvement of nursing and midwifery staff in decision‐making rather than shared interdisciplinary responsibility.

Today, I saw numerous neonatologists and residents facilitating discharges and offering counselling to the mothers. Nursing is notably lacking in this context.

(NUR04)

(…) Typically, the discharge is given by the doctors. We receive a document indicating that the mother and infant are being discharged.

(NUR05)

Here, the doctors give the obstetric discharge and then the neonatologist has to determine whether the baby will be discharged or not.

(NUR06)

Look, I think the one who gives the discharge is mainly the doctor. (…) It's them who should give the main counselling.

(NUR08)

### 4.3 Leadership and Shared Information About Healthcare Practices

Despite references to multiple professionals, participants' accounts revealed fragmented leadership and inconsistent information sharing rather than coordinated interdisciplinary practice.

The doctors give information on scheduling follow‐up appointments, prescribing medications. And the nursing staff mainly provides on how to give the bath, how the suction is during breastfeeding. The psychologist, depending on the case, also participates.

(D04)

The nursing technician also provides the same counselling, and I (nurse‐midwife) just sign the discharge along with the obstetrician and the neonatologist.

(MID05)

(…) some nurse‐midwives participate (in the moment when counselling is given), we technicians also participate, but I think less so.

(NUR08)

It has been reported that nurse‐midwives focus their caring for mothers on management of surgical wounds and breast care.

I try to give support, emphasizing care for the surgical wound…to prevent infection.

(MID01)

I try to check how the breast is… I give advice on massage, to avoid using hot water.

(MID02)

Although exclusive breastfeeding was consistently framed as the preferred and dominant practice, several participants also described providing guidance on formula preparation when needed, revealing a tension between normative expectations and practical care realities.

I try to focus more on breastfeeding. We cannot talk necessarily about how to do the bath, but breastfeeding is essential.

(MID01)

Our role here is to always guide the mother regarding baby care, and basically, the focus is exclusive breastfeeding…here, we are very focused on the issue of breastfeeding.

(NUR03)

We provide counselling on feeding, which is exclusive breastfeeding at this time.

(MID03)

I think that talking about breastfeeding it's fundamental, because we see a very high risk of newborn readmission…

(D05)

### 4.4 Transition From Hospital Care to Care at Home

These findings reflect the specific characteristics of the rooming‐in setting, where discharge preparation is embedded in daily care and develops through continuous contact between mothers, newborns, and healthcare professionals. Participants described discharge preparation as a longitudinal process; however, this occurred in the absence of a structured or standardized discharge pathway.

She (the mother) should avoid crowded places, avoid visits from people who are sick or have a cold, and, in general, be careful with hand washing and hygiene.

(MID01)

The most important is […] advice regarding the bath and the care for the umbilical cord.

(NUR02)

At home, in case of any signs of abnormality, she (the mother) should seek healthcare services, contact the emergency unit.

(NUR03)

The nursing team also does its part by reinforcing counselling on sunbathing, care for the diaper changes, breastfeeding position, burping the baby, sleeping position, and others.

(MID03)

Umbilical cord cleaning is partially explained during professional shifts, but then we reinforce immediate care instructions, like baby choking, what to do, right?

(MID04)

I provide guidance on vaccinations, clarify doubts regarding baby care or procedures, and that's it.

(NUR08)

The team expresses worries regarding the scheduling of follow‐up appointments and referrals to healthcare services in relation to the neonatologist's participation in newborn care.

We give a referral document to the health clinic, fill out the health booklet, and provide this instruction for follow‐up procedures. Then, when handing it over, we usually have a quick conversation about the basic guidance, which is what's in that booklet we give.

(D01)

Referral to the hospital, yes, women were informed about the breastfeeding services in the post‐partum normally, that's what we always refer to, and also we prescribe vitamin D.

(D02)

Provides counselling on what the baby will need at home and the follow‐up appointments.

(D03)

Furthermore, the team provides information on fundamental and particular care that mothers should provide to newborns at home alone at the time of hospital discharge, perhaps creating the process less comprehensible. The assertions emphasize this aspect:

Babies who are discharged with infant formula supplementation, we guide them on how to prepare it; those are the main points.

(D01)

Child's feeding, warning signs to seek emergency medical care, and what should not be done with the baby at home.

(D03)

I explain all the care they need to have at home, like preventing sudden infant death, what should not be used on the baby.

(D04)

The mother receives a flyer with instructions… warning signs for the baby, fever, low activity, an inconsolable crying, cyanosis, difficulty breathing, the correct sleeping position, regarding sudden infant death…how to prepare the formula […], medication usage.

(D05)

This category shows that for healthcare professionals, the transition from hospital to home is an ongoing process experienced throughout the hospitalization, rather than merely at the point of discharge.

Because in rooming‐in, from the first day you admit the mother, you are providing information to her about caring for the baby.

(MID02)

Yes, I think the hospital discharge is a daily preparation, starting from birth, from the delivery room, with daily information, daily visits, and conversations with the mother. So, discharge begins with the admission process…

(D05)

Nonetheless, professionals acknowledge the lack of a systematic and well‐defined discharge plan of action.

Look, I understand that here, discharge doesn't have a standard procedure.

(NUR08)

There is a lack of a proper discharge routine for the moment when the mother and baby are discharged […], but not just at the moment when she is receiving all the papers and about to leave through the door.

(MID02)

I'm not sure if there is a setting where nursing professionals participate, I don't know if it's just nurse‐midwives and doctors, I've never participated.

(NUR03)

I think all this information she has already been receiving during the hospitalization could be better reinforced when she's about to be discharged.

(MID04)

## 5 Discussion

The transition from hospital to home represents a critical phase in newborn care and relies on a coordinated relationship between healthcare professionals and families at the time of discharge of the mother‐infant dyad. Parental readiness is widely recognized as a crucial component of discharge procedures and represents a key consideration guiding healthcare professionals' discharge planning and counseling practices. Ingram et al. [29] describe this transition as extending beyond clinical stability to include emotional support, education, and continuity of care, all of which are most effectively delivered through a multidisciplinary approach. Accordingly, hospital discharge is conceptualized in the literature as a multifaceted and longitudinal process that should begin at admission and be supported by effective communication, shared responsibility, and structured planning across professional groups [30]. These findings can also be interpreted in light of established conceptual frameworks on care transitions and discharge readiness, which conceptualize discharge as a coordinated, multidimensional process requiring effective communication, shared responsibility, and continuity of care across settings [22, 23]. In this context, the rooming‐in model plays a central role, as it transforms discharge preparation into a continuous and relational process rather than a discrete, time‐limited intervention.

In contrast to these recommended models, the findings of the present study reveal a discharge process that remains predominantly physician‐led, with limited multidisciplinary collaboration. Participants consistently described doctors as the primary decision‐makers for newborn discharge and, in some cases, as the main providers of parental counseling. This is particularly relevant in rooming‐in settings, where continuous proximity to mothers and newborns would theoretically support greater involvement of nursing and midwifery professionals in discharge preparation. Nursing and midwifery professionals, despite their continuous presence in the RIU, were often described as marginally involved in final discharge decisions. This pattern highlights a persistent medicalization of discharge practices and reflects organizational and cultural barriers that constrain shared decision‐making, despite the acknowledged importance of multidisciplinary collaboration [31]. The limited integration of nurses and midwives in discharge planning is particularly significant given their established role in postnatal care. Nurses and midwives are key caregivers during and after birth, with responsibilities that include clinical assessment, parental education, and coordination of care. They frequently act as the first point of contact for families and are well‐positioned to assess parental readiness and reinforce discharge education [32]. Evidence suggests that targeted educational initiatives substantially improve midwives' knowledge and competence in newborn care, contributing to safer discharge practices [33]. Nevertheless, the findings of this study indicate a discrepancy between these professional competencies and their actual involvement in discharge decision‐making, underscoring a gap between professional roles as defined in the literature and their enactment in clinical practice.

Infant feeding emerged as a central component of discharge counseling across professional groups. This emphasis aligns with existing evidence highlighting the role of nurses and midwives in supporting breastfeeding as a cornerstone of neonatal care and discharge planning [34]. Human milk is widely recognized as the optimal source of nutrition for newborns, offering substantial benefits for both infants and mothers [35]. Participants frequently described breastfeeding as the primary focus of discharge education and as a key strategy for promoting neonatal health and preventing readmissions.

At the same time, the findings revealed important tensions between normative breastfeeding discourse and the realities of clinical practice. While exclusive breastfeeding was consistently promoted as the preferred and expected standard, several participants reported providing guidance on formula preparation and alternative feeding strategies when clinically or socially indicated. These accounts suggest that discharge counseling often occurs within a framework of strong professional and cultural expectations surrounding exclusive breastfeeding, which may limit open discussion of individualized feeding needs. Rather than reflecting inconsistency in professional knowledge, this tension highlights the complexity of infant feeding counseling at discharge and underscores the need for structured, family‐centered approaches that balance evidence‐based breastfeeding promotion with clear, non‐stigmatizing guidance on alternative feeding options [36].

Beyond infant feeding, participants described broader deficiencies in the structure and organization of discharge preparation. Essential information related to home hygiene, recognition of warning signs, safe sleeping positions, and follow‐up care was often delivered in a fragmented and inconsistent manner [37]. Although discharge preparation was described as a longitudinal process occurring throughout hospitalization, it lacked formal structure and standardization. This fragmentation may compromise parental understanding and preparedness, increasing the risk of adverse outcomes after discharge. Inadequate post‐discharge care has been directly linked to unplanned readmissions and neonatal mortality [4, 38], many of which are associated with preventable factors.

Despite these challenges, healthcare professionals acknowledged the importance of planning and expressed awareness of the need for more structured discharge processes. The literature strongly supports the standardization of discharge protocols as a means to enhance patient safety, reduce variability in professional practices, and improve continuity of care [39]. Personalized discharge planning, combined with systematic assessment of newborn health and parental readiness, has been shown to reduce infection risk and emergency readmissions [40]. In contrast, the findings of this study suggest that such practices are not yet consistently embedded in routine care within the rooming‐in setting examined.

Overall, this study highlights a clear discrepancy between evidence‐based recommendations for multidisciplinary discharge planning and the realities reported by healthcare professionals. Rather than illustrating effective interdisciplinary collaboration, the findings reveal structural, cultural, and organizational constraints that limit shared responsibility, coordinated education, and comprehensive discharge preparation. Addressing these gaps will require not only the implementation of standardized tools and checklists but also organizational and cultural changes that support genuinely collaborative, family‐centered discharge practices aligned with current evidence.

### 5.1 Strengths and Limitations

The research offers multidisciplinary perceptions on newborn discharge in Brazil. Multidisciplinary teams are directly involved in the process of transitioning newborns from hospital to care at home. Every procedure done is responsible for the entire family's well‐being. One strength of this study is underscoring the need of building interdisciplinary teams within RIU. The scientific contribution also advocates for the establishment of standardized care practices nationwide, according to WHO international recommendations.

Nonetheless, a weakness of this study is that it included only three groups of healthcare professionals within the care unit. The lack of a comprehensive multidisciplinary team, comprising a nutritionist, psychologist, social workers, and other care providers during rooming‐in, may constitute a limitation of the study. The perspectives of these disciplines involved on a consultative or case‐specific basis were not explored, but should be considered in future research.

Additionally, the study was conducted during the COVID‐19 pandemic, which may have influenced care processes, staffing dynamics, and discharge practices. This contextual factor should be considered when interpreting the findings.

### 5.2 Recommendations for Future Research

Future research could investigate the creation of a cultural‐specific checklist to facilitate the discharge of the infant; it is required that all subjects discussed during the counseling session have been thoroughly addressed. At that juncture, the team releasing the family feels confident that all subjects potentially provoking parental anxiety or concerns have been addressed.

### 5.3 Implications for Policy and Practice

The hospital discharge process necessitates further growth and the engagement of the healthcare team, especially in formulating methods that effectively tackle the issue and positively influence the infant and their family. This research can inform the development of future protocols for healthcare facilities and aid healthcare professionals in delivering appropriate guidance to families. Moreover, the results can enhance nursing care from hospitalization to the transition to care at home.

Finally, it is fundamental that healthcare organizations prioritize the standardization of training in maternal and neonatal counseling. During a critical period for the family, such as the arrival of a newborn, it is important that all professionals standardize information in a consistent and universal manner. The ongoing education of midwives, doctors, nurses, and other healthcare professionals is essential for optimal health outcomes for the entire community.

## 6 Conclusion

This study identifies a clear gap between evidence‐based recommendations for multidisciplinary discharge planning and the practices reported by healthcare professionals in a RIU. The findings highlight a predominantly physician‐led discharge process, limited interdisciplinary involvement, and fragmented approaches to parental education, which may compromise parental readiness during the transition from hospital to home.

The results underscore the importance of strengthening the role of nursing professionals, particularly nurses and nursing technicians, in discharge planning and counseling. Given their continuous presence in the unit and their central role in education and care coordination, greater integration of nursing staff may enhance family‐centered care and continuity across the discharge process.

Infant feeding counseling emerged as a complex and sensitive component of discharge preparation. While exclusive breastfeeding was strongly promoted, participants also described the need to provide guidance on alternative feeding methods in specific circumstances. These findings emphasize the need for clear, balanced, and non‐stigmatizing feeding counseling that reflects both evidence‐based recommendations and real‐world care needs.

Overall, the study suggests that greater structure and standardization of newborn discharge processes are needed. The implementation of evidence‐based discharge protocols, supported by validated checklists and clear educational materials, may reduce variability in practice, promote interdisciplinary collaboration, and improve the quality and safety of post‐hospitalization care for the mother‐ infant dyad. In rooming‐in settings, where discharge preparation is inherently embedded in daily care, strengthening multidisciplinary collaboration is essential to fully realize the potential of this care model.

## Research Team

T.E. (MSc, PhD Student, Neonatology Nurse), A.R. (Professor in Public Health), and M.H. (Professor in Public Health) were responsible for the conceptualization and design of this study. The data collection was conducted by T.E. The data analysis and interpretation were carried out by T.E. and C.L. (MSc, PhD Student, Midwife), who drafted the initial manuscript. A.N. (PhD, Research Midwife) and S.F. (PhD, Research Midwife) critically reviewed, revised the manuscript, and approved the final version for publication. All authors take responsibility for the integrity of the data and the accuracy of the data analysis. The corresponding author attests that all listed authors meet authorship criteria and that no others meeting the criteria have been omitted.All authors have read and approved the final version of the manuscript. CL, as the corresponding author, had full access to all of the data in this study and takes complete responsibility for the integrity of the data and the accuracy of the data analysis.

## Author Contributions

**Thaís Emanuele da Conceição:** conceptualization, methodology, investigation, data curation, writing – original draft. **Adriana Duarte Rocha:** conceptualization, methodology. **Cristina Lumia:** writing – original draft, software, formal analysis. **Simona Fumagalli:** methodology, writing – review and editing, data curation. **Antonella Nespoli:** conceptualization, supervision. **Maria Helena do Nascimento Souza:** conceptualization, supervision.

## Funding

The authors have nothing to report.

## Conflicts of Interest

The authors declare no conflicts of interest. All co‐authors have seen and agree with the contents of the manuscript, and there is no financial interest to report. We certify that the submission is original work and is not under review at any other publication.

## Transparency Statement

The lead author Cristina Lumia affirms that this manuscript is an honest, accurate, and transparent account of the study being reported; that no important aspects of the study have been omitted; and that any discrepancies from the study as planned (and, if relevant, registered) have been explained.

## Supporting information

Supporting File

## Data Availability

Data are available from the corresponding author upon reasonable request. The data that support the findings of this study are available from the corresponding author upon reasonable request.
