# Supplementary material for: Multidisciplinary Team Perceptions on Preparing Newborns for Discharge From a Rooming‐in Unit: A Qualitative Study
Source: Health Sci Rep. 2026 Apr 27;9(5):e72422. doi: 10.1002/hsr2.72422 (PMC13112597; doi:10.1002/hsr2.72422)
Supplement: Supplementary file 1 — Supporting File [file HSR2-9-e72422-s001.docx]

**Supplementary Material 1.**

**Semi-structured interview guide**

**Section A - Professional role and involvement**

1. Can you describe your professional role in the rooming-in unit?
2. What is your involvement in the preparation of mothers and newborns for hospital discharge?

**Section B - Responsibility and decision-making**

1. How is the decision to discharge a newborn usually made in this unit?
2. Which professionals are involved in this decision, and how are responsibilities distributed?
3. In your experience, do all team members have the opportunity to contribute to discharge decisions? Give an example, please.

**Section C - Information sharing and parental counselling**

1. What type of information or counselling is usually provided to parents before discharge?
2. Which professionals provide this information, and on which topics?
3. Are there aspects of counselling that are emphasized more than others?
4. How is information shared or coordinated among different professionals?

**Section D - Transition from hospital to home**

1. How do you perceive the transition from hospital care to care at home for newborns and families?
2. What guidance is given to parents regarding home care, warning signs, and follow-up services?
3. In your opinion, is discharge preparation a single moment or an ongoing process during hospitalization?

**Section E - Challenges and improvements**

1. What challenges do you encounter during the discharge process?
2. Do you think the discharge process is well standardized?
